# Experimental and Computational Analysis of Polyglutamine-Mediated Cytotoxicity

**DOI:** 10.1371/journal.pcbi.1000944

**Published:** 2010-09-23

**Authors:** Matthew Y. Tang, Carole J. Proctor, John Woulfe, Douglas A. Gray

**Affiliations:** 1 Ottawa Hospital Research Institute, Ottawa, Ontario, Canada; 2 Department of Biochemistry, Microbiology and Immunology, University of Ottawa, Ottawa, Ontario, Canada; 3 Centre for Integrated Systems Biology of Ageing and Nutrition, Institute for Ageing and Health, Newcastle University, Newcastle upon Tyne, United Kingdom; EMBL Heidelberg, Germany

## Abstract

Expanded polyglutamine (polyQ) proteins are known to be the causative agents of a number of human neurodegenerative diseases but the molecular basis of their cytoxicity is still poorly understood. PolyQ tracts may impede the activity of the proteasome, and evidence from single cell imaging suggests that the sequestration of polyQ into inclusion bodies can reduce the proteasomal burden and promote cell survival, at least in the short term. The presence of misfolded protein also leads to activation of stress kinases such as p38MAPK, which can be cytotoxic. The relationships of these systems are not well understood. We have used fluorescent reporter systems imaged in living cells, and stochastic computer modeling to explore the relationships of polyQ, p38MAPK activation, generation of reactive oxygen species (ROS), proteasome inhibition, and inclusion body formation. In cells expressing a polyQ protein inclusion, body formation was preceded by proteasome inhibition but cytotoxicity was greatly reduced by administration of a p38MAPK inhibitor. Computer simulations suggested that without the generation of ROS, the proteasome inhibition and activation of p38MAPK would have significantly reduced toxicity. Our data suggest a vicious cycle of stress kinase activation and proteasome inhibition that is ultimately lethal to cells. There was close agreement between experimental data and the predictions of a stochastic computer model, supporting a central role for proteasome inhibition and p38MAPK activation in inclusion body formation and ROS-mediated cell death.

## Author Summary

Neurodegenerative diseases feature concentration of misfolded or damaged proteins into inclusion bodies. There is controversy over whether these entities are protective, detrimental, or relatively benign. The formation of inclusion bodies may be accelerated by inefficient protein degradation and may promote activation of stress signalling pathways. Each of these events may promote the generation of reactive oxygen species which may exacerbate the problem by damaging more proteins, possibly damaging components of the UPS itself, but in either case further impeding the function of cellular proteolytic systems. To determine how these events are related and which are critical, we generated a live cell imaging system in which inclusion formation and proteolytic efficiency can be evaluated, and created a stochastic computer model incorporating the same components. Laboratory data and computer simulations were found to be in close agreement, supporting a mechanism wherein misfolded protein induced a vicious cycle of stress kinase activation, ROS generation, and proteasome inhibition which was ultimately cytotoxic. Inclusion body formation partially alleviated the burden on the proteolytic system, but may not provide long term benefit. Pharmacological blockade of a stress-activated kinase was effective in breaking the vicious cycle, as predicted by the computer model and confirmed experimentally.

## Introduction

A hallmark feature of human neurodegenerative diseases is the accumulation of misfolded or otherwise abnormal proteins which become concentrated into large aggregates. Inclusion bodies are large nuclear or cytoplasmic protein aggregates whose predominant constituents may be characteristic of particular diseases. In many cases inclusion bodies (IB) are immunoreactive for ubiquitin and proteasome components [1], indicative of abortive or incomplete proteolysis. The sustained expression of mutant protein with the propensity to misfold may ultimately overwhelm the ubiquitin/proteasome system (UPS) and promote the formation of inclusions. This process may be accelerated by an age-related decline in UPS efficiency (discussed in [2]), which may explain why genetically transmitted neurodegenerative disorders typically affect older individuals. Consistent with the proteasome impairment hypothesis, IB form in the neurons of mice in which proteasome function has been genetically compromised [3]. Because misfolded, damaged, or genetically abnormal proteins are aggregation-prone their sequestration into inclusion bodies may actually alleviate the load on the UPS and promote neuronal survival, at least in the short term. Time lapse microscopy of a fluorescent proteasome reporter in cultured neurons has indicated that the UPS load is partially alleviated upon IB formation [4], and there is evidence that cultured cells forming such inclusions have a survival benefit [5] over the course of the experiment. In the longer term, however, it is possible that deleterious effects from IB formation would become pronounced. Apart from potential physical perturbations imposed by large proteinaceous inclusions (in axons, for example) these entities may wreak havoc by depleting essential cellular components (reviewed in [6]) or by biochemical means. In Huntington's disease, IB form when a polyglutamine tract in the N-terminal region of the huntingtin protein exceeds the threshold length of approximately forty glutamine residues; early onset and severe disease are correlated with very long tracts, whereas huntingtin proteins with polyglutamine tracts shorter than the threshold do not form IB and are not pathogenic [7]. The nuclear IB formed by the mutant huntingtin protein are generators of reactive oxygen species [8], and expression of such an expanded polyglutamine protein results in sustained and ultimately cytotoxic activation of p38MAPK [9]. It is likely that proteasome inhibition, ROS generation, and p38MAPK activation all feature in the death of cells containing IB, but their relative importance and potentially complex interdependencies are poorly understood. We have combined live cell imaging with mathematical modeling to explore such relationships. Our data point to a positive feedback loop between IB formation and p38MAPK activation that likely involves ROS. The existence of this loop is supported by the close agreement of laboratory data and simulations generated by a stochastic computer model.

## Results

### p38MAPK inhibition rescues cells from polyglutamine-induced cell death

**Figure 1:**
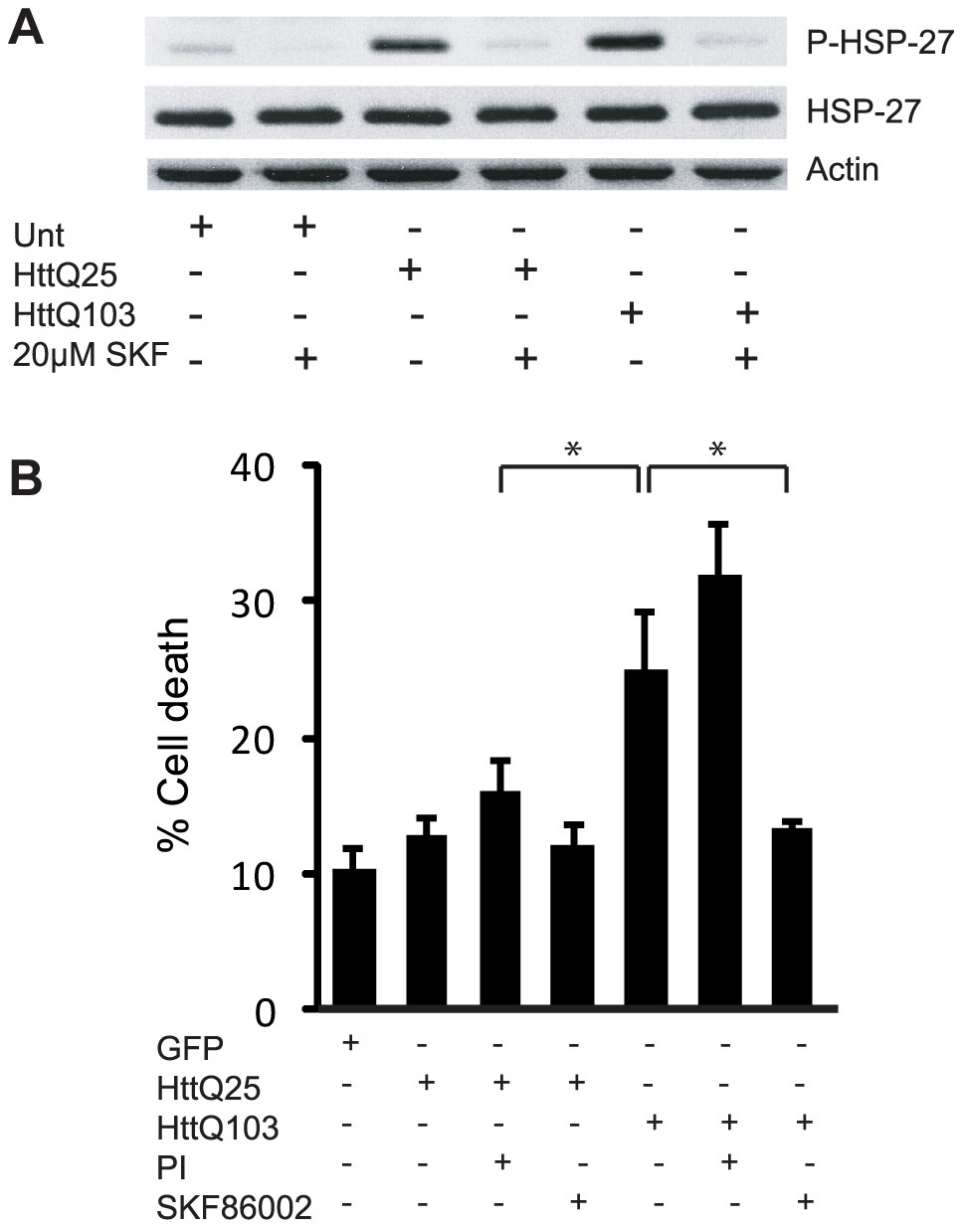
SKF86002 rescues cells from expanded-polyglutamine induced cell death. A) Western blot analysis with an antibody specific for phospho-HSP-27 in extracts from U87MG cells expressing HttQ25 or HttQ103. Cells were untreated or were treated with the p38MAPK inhibitor SKF86002. Expression of expanded HttQ103 activated p38MAPK and resulted in phosphorylation of HSP-27, a downstream target of p38MAPK. The analysis revealed a significant reduction of HSP-27 phosphorylation in HttQ25 and HttQ103 expressing cells treated with SKF86002. Re-probing the membrane with an antibody raised against total-HSP-27 revealed no significant difference in the total levels of HSP-27 protein. Actin served as a loading control. B) Cell viability of U87MG cells expressing GFP, HttQ25 or HttQ103 treated with proteasome inhibitor (PI) or p38MAPK inhibitor (SKF86002) was assessed by flow cytometry analysis using propidium iodide exclusion. The survival of SKF86002 treated HttQ103 expressing cells was significantly improved when compared to untreated counterparts. Treatment with PI resulted in a significant increase in cell death in cells expressing HttQ103 when compared to HttQ25 expressors. Error bars indicate the standard error of the mean (*p<0.05).

We have previously demonstrated sustained activation of p38MAPK in cultured mammalian cells expressing expanded polyglutamine proteins and in a transgenic mouse model of expanded-polyglutamine disease [9]. This activation could be abrogated by treatment with the specific p38MAPK inhibitor SKF86002. To confirm the activation of p38MAPK and its inhibition by SKF86002 we performed western blot analysis of U87MG cells expressing HttQ103 (an amino terminal fragment of the human huntingtin protein containing a 103 glutamine tract). Expression of the expanded polyglutamine protein resulted in extensive phosphorylation of HSP-27, a downstream target of p38MAPK (Figure 1A). Reduced phosphorylation of HSP-27 was detected in cells expressing HttQ25 and HSP-27 phosphorylation was undetectable in untransfected control cells. Although the HttQ25 protein contains a polyglutamine tract below the threshold length for pathogenicity, its overexpression upon transfection may be sufficient to activate p38MAPK at a low and nontoxic level as we have documented previously [9]. The phosphorylation of HSP-27 was precluded by pre-treatment of transfected cells with a pharmacological inhibitor of p38MAPK (Figure 1A) or by co-expression with a dominant-negative (kinase dead) variant of p38MAPK (Figure S1) confirming that the phosphorylation of HSP-27 was due to the activation of the p38MAPK pathway by Htt103.

To determine if blockade of p38MAPK activity affects cell survival, we transfected U87MG cells with either HttQ25, HttQ103 or a GFP control plasmid. At 30 hours post-transfection, flow cytometry was performed using propidium iodide exclusion, and revealed that HttQ103 expressing cells exhibited the highest levels of death (25%, Figure 1B) whereas the death associated with Htt25 expression was similar to that of GFP (10–12%). Pre-treatment of HttQ25 and HttQ103cells with SKF86002 resulted in a decrease in cell death that was most pronounced in cells expressing HttQ103 (Figure 1B). The HttQ103 protein is known to inhibit proteasome activity in a cell-based assay [10]; to determine if the cytotoxicity of the expanded-polyglutamine proteins could be enhanced by further proteasome inhibition a pharmacological proteasome inhibitor (PI) was added to HttQ25 and HttQ103 transfected cells. Cells were pre-treated with PI for 6 hours prior to assessing cellular death by flow cytometry. PI-treated U87MG cells expressing HttQ103 exhibited a significant increase in cell death when compared to their untreated counterparts (Figure 1B), and was 15% greater than PI treated HttQ25 expressing cells. Under the same experimental conditions the amount of cell death induced by PI in untransfected cells was approximately 5% (not shown), roughly equivalent to the increase in cell death mediated by PI in cells expressing HttQ25 and HttQ103. Under our conditions proteasomes cannot therefore be fully inhibited by expression of the polyQ proteins alone. The pharmacological data support the argument that the cytotoxicity of misfolded proteins is mediated by proteasome inhibition and p38MAPK activation, but do not reveal whether these activities are independent.

### IB formation is promoted by p38MAPK activity, and proteasome dysfunction

**Figure 2:**
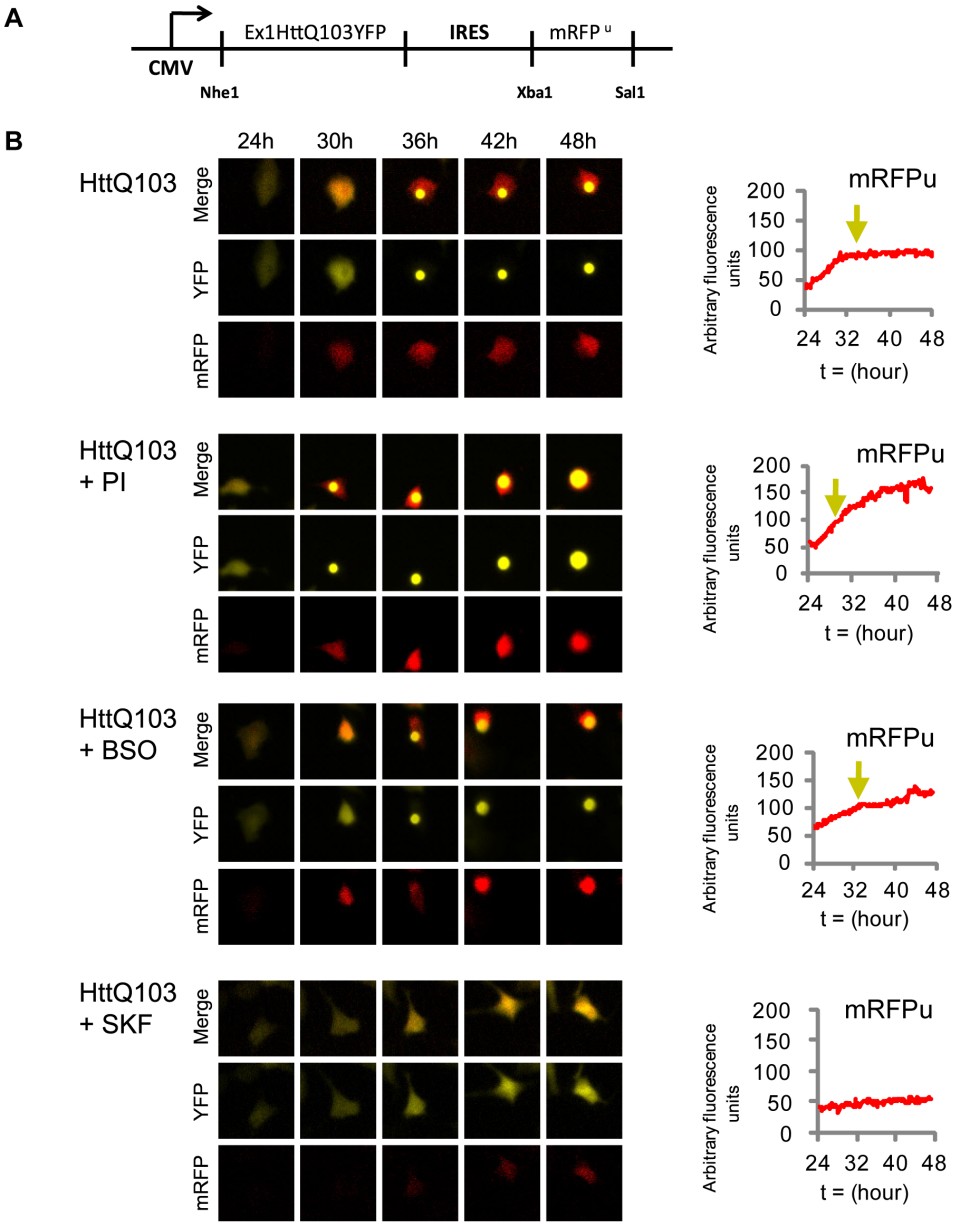
Expression of HttQ103 results in the formation of inclusion bodies (IB) that are preceded by increased levels of the proteasome reporter protein mRFP^u^. A) Schematic representation of the bicistronic expression construct engineered to simultaneously express the huntingtin-derived protein HttQ103 fused to the yellow fluorescent protein (YFP) and an intrinsic proteasome activity reporter (mRFP^u^) downstream of an internal ribosome entry site (IRES). B) Single cell analysis of IB formation and UPS impairment induced by the expression of the HttQ103 protein. U87MG cells expressing HttQ103YFP-pIRES-mRFP^u^ were imaged over the course of 24 hours to simultaneously follow IB formation and UPS impairment. Cells were either left untreated (panel 1) or treated with proteasome inhibitor (panel 2), buthionine sulphoximine (BSO, panel 3) or the p38MAPK inhibitor (SKF86002, panel 4) and visualized under fluorescence at 10 minute intervals. Over time accumulation of mRFP^u^ was graphed using densitometry values from a single cell for each condition which revealed detectible levels of mRFP^u^ prior to the formation of an IB (represented by the yellow arrow). Treatment with PI resulted in IB formation at an earlier time-point and correlated with increased levels of mRFP^u^. BSO treatment also increased mRFP^u^ levels but did not significantly accelerate the timing of IB formation. Treatment with SKF86002 resulted in overall lower levels of mRFP^u^ and a delay in inclusion body formation. Single cells were isolated from the population of cells described in Figure 3.

To simultaneously assess functioning of the UPS and inclusion body (IB) formation at a single cell level we created a bicistronic construct that encodes an expanded polyglutamine protein and fluorescent proteasome substrate on the same transcript (see schematic diagram in Figure 2A). This construct was designed to investigate the temporal order of events leading to HttQ103 induced cellular and proteasome toxicities and to dissect the role of p38MAPK in these events.

U87MG cells were transfected with HttQ103YFP-pIRES-mRFP^u^ and were imaged at 10 minute intervals from 24 to 48 hours post-transfection. The time lapse images revealed formation of IB at 36 hours post-transfection in cells expressing HttQ103 (Figure 2B, first panel; time lapse videos are provided as Videos S1 to S8). Values of mRFP^u^ intensity were graphed as a function of time revealing an increase in mRFP^u^ fluorescence prior to the formation of an IB, followed by a period of constant mRFP^u^ intensity. The single cell analysis revealed that HttQ103-induced cellular death is preceded by gradual UPS impairment. Once this impairment reaches a threshold level, IBs begin to form and their formation correlates with a momentary recovery of UPS efficiency as measured by mRFP^u^ intensity. These results are consistent with previously published findings in primary neuron cultures [4].

To examine the extent to which IB formation was dependent on UPS dysfunction U87MG cells expressing HttQ103YFP-pIRES-mRFP^u^ were treated with proteasome inhibitor. As expected PI treatment resulted in a rapid and persistent increase in mRFP^u^ intensity. Under these conditions IB formation was accelerated relative to untreated controls (Figure 2B, second panel). These data suggest an immediate relationship between proteasomal inhibition and IB formation.

Damaged proteins are normally eliminated by the UPS, and we speculated that an increase in cellular ROS levels would lead to oxidative damage to proteins and inflict an additional burden on proteasomes that may affect the kinetics of IB formation. To test this hypothesis, we depleted reduced glutathione levels in HttQ103YFP-pIRES-mRFP^u^ tranfected cells by treating cells with buthionine sulphoximine (BSO) at 24 hours post-transfection. In BSO treated cells we observed a constant increase in mRFP^u^ intensity (Figure 2B, third panel) consistent with a cumulative UPS burden.

Having previously established that the activation of p38MAPK in HttQ103 expressing cells contributes to cytotoxicity, we sought to determine what effects inhibition of p38MAPK signalling pathway would have on IB formation and UPS dysfunction. U87MG cells expressing HttQ103YFP-pIRES-mRFP^u^ were therefore treated with SKF86002. These cells exhibited a low level of mRFP^u^ fluorescence along with a delay in IB formation (Figure 2B, fourth panel). The mRFP^u^ fluorescence remained low and did not feature a rapid increase as seen in the untreated counterparts. These data suggest that inhibition of the p38MAPK pathway decouples the proteasome inhibition from HttQ103 protein expression, resulting in delayed formation of IB.

**Figure 3:**
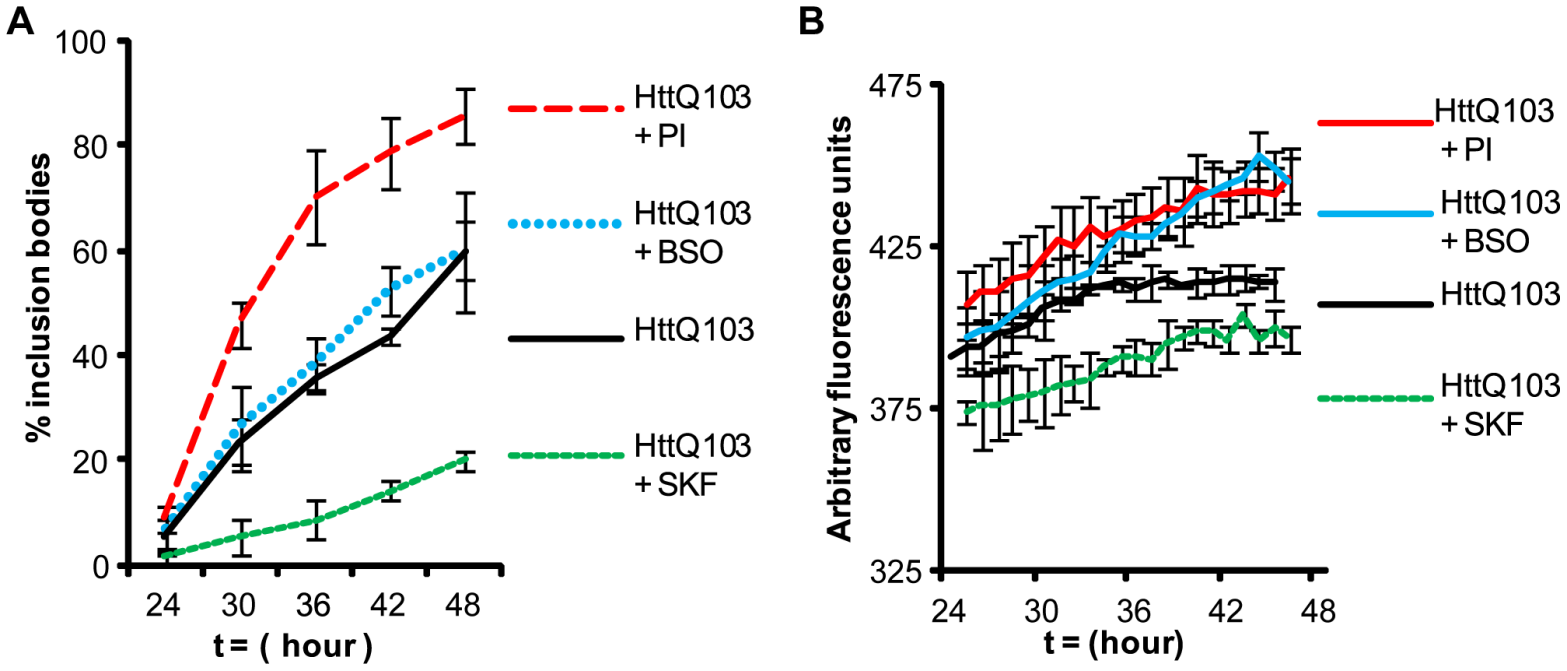
Data from multiple live cell analyses of IB formation and proteasome inhibition. A) The percentage of IB formation for each treatment was recorded every 6 hours beginning at 24 hours post-transfection. B) Average mRFP^u^ intensity values for multiple cells over time. Experiments were performed in triplicate with over 50 cells analyzed in each condition. Error bars indicate standard error of the mean.

We quantified IB formation by recording the number of cells with IB at 6 hour intervals starting at 24 hours post-transfection. The percentage of cells with IB was graphed as a function of time (Figure 3A). PI treatment generated the greatest number of IB compared to untreated cells while SKF86002-treated cells were found to have the fewest IB. Comparative single-cell analysis of IB formation corresponded to time-points in which 40% of transfected cells had formed IB. Similarly, we quantified average mRFP^u^ fluorescence from many cells and graphed the fluorescence intensities as a function of time. Treatment with PI and BSO generated the highest amounts of mRFP^u^ fluorescence, while SKF86002 treatment resulted in the lowest levels of mRFP^u^ in comparison to untreated cells (Figure 3B). Findings from multiple cells were consistent with the mRFP^u^ fluorescence levels observed in the single cell analysis.

### The critical role of p38MAPK is supported by mathematical modeling

**Figure 4:**
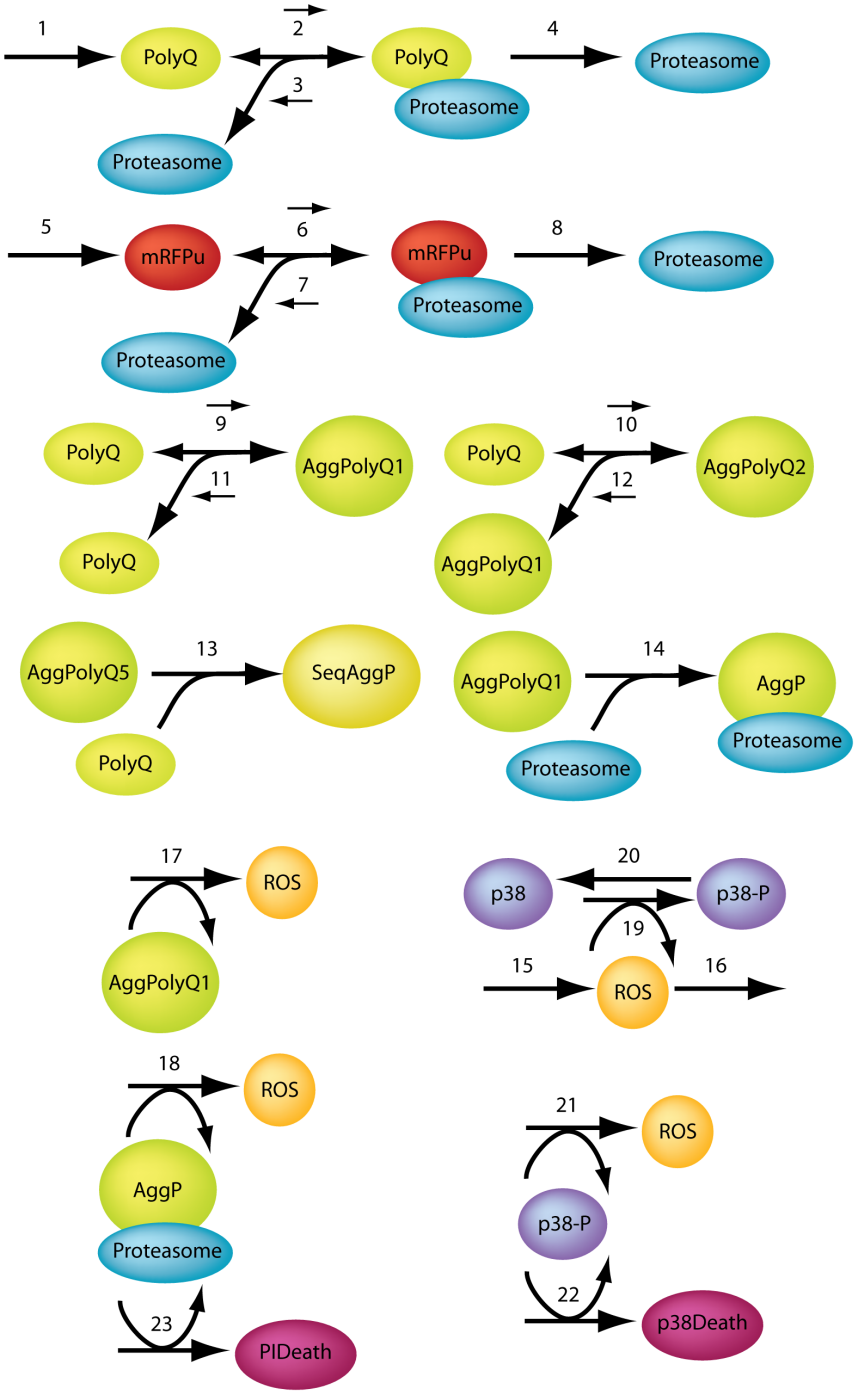
Diagram of molecular relationships simulated in the computer model. The model assumptions are presented in Text S1, along with lists of molecular species (Table S1 in Text S1) and reaction parameters (Table S2 in Text S1).

**Figure 5:**
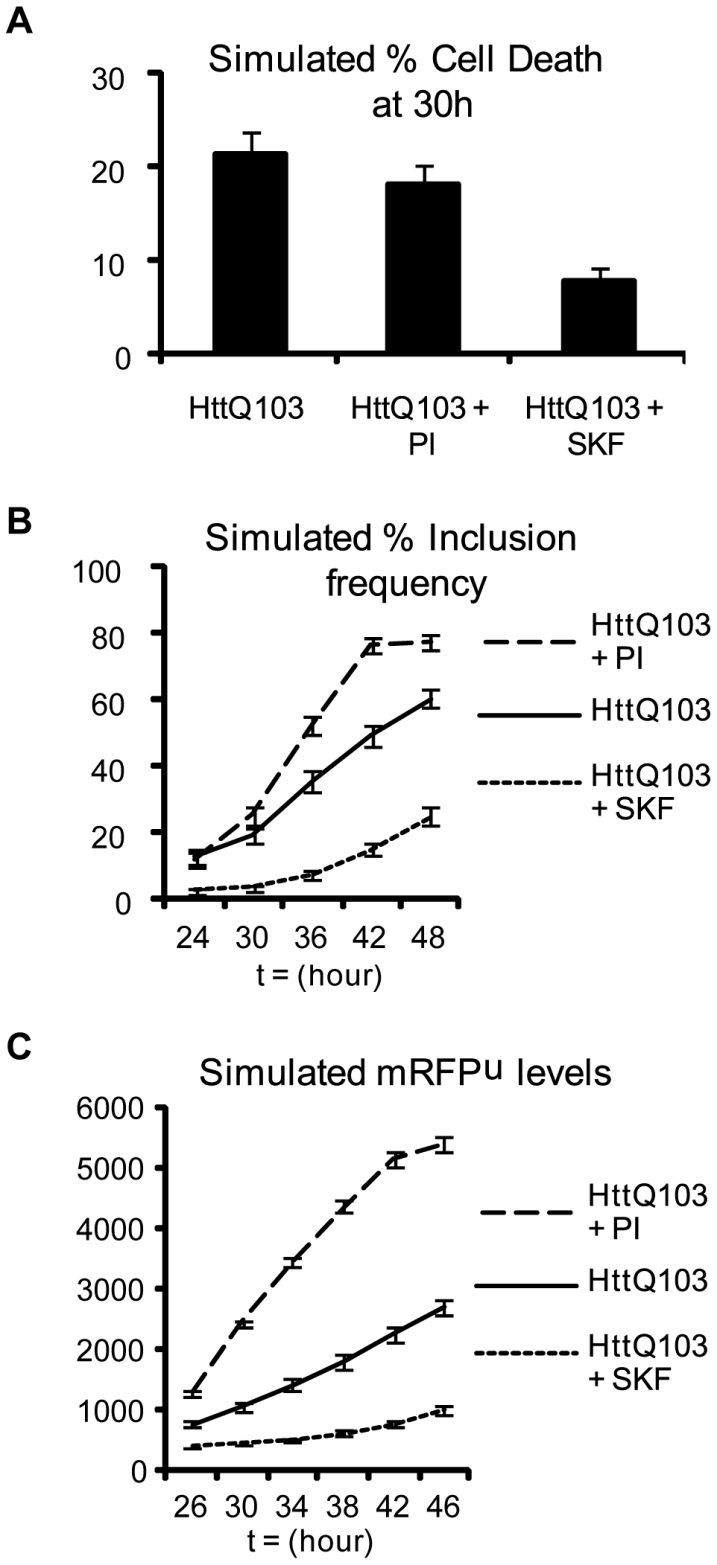
Simulations generated by a stochastic computer model of polyglutamine-mediated cytotoxicity. A) Model predictions for cell death at 30 hours. Results show percentage of cells that died by 30 hours in 300 simulations of each computer experiment. B) Model predictions for kinetics of inclusion formation. Results show percentage of cells with inclusions at each time point from 300 simulations for each computer experiment. C) Model predictions for accumulation of mRFP^u^. The mean level of mRFP^u^ for 300 simulations of each computer experiment was calculated for each of the time-points shown. The y-axis shows the number of molecules predicted by the simulations. Error bars for the model predictions represent standard error of the mean.

We have previously used stochastic computer modeling to study the age-related decline of proteolysis [11], and have adapted this model to incorporate p38MAPK in an effort to better understand polyglutamine-mediated cell death. Our objective was to determine if a relatively simple mathematical model incorporating the components thought to be critical for polyQ-mediated cytotoxicity could recapitulate our laboratory findings; if not some critical component must have been overlooked or one or more of the starting assumptions must be invalid. Conversely, a good fit would suggest that the assumptions are valid and no critical elements have been overlooked. The stochastic computer model is represented schematically in Figure 4. The model was constructed using the Systems Biology Markup Language as described in the Materials and Methods section; details of molecular species and reactions are given in Text S1. The model predicted that treatment with PI would lead to reduced cell death at 30 hours (Figure 5A), a short term benefit from reduced levels of small aggregates binding to proteasomes (the consequence of which would be reduced by concentration of aggregates into IB). The experimental data, however, indicated a slight increase in the proportion of cell deaths under PI suggesting that the situation is more complex than is currently accounted for in the model. On the other hand, the model predicted that inhibition of p38MAPK activity should lead to much lower cell death, in close agreement with the experimental data. Also in agreement with the experimental findings described above, the model predicted that proteasome inhibition should lead to an increase in the rate of IB formation compared to untreated cells (Figure 5B). When p38MAPK activity is inhibited the model predicts a lower rate of IB formation at early time-points (Figure 5B) although from 36–48h, the rate of increase is similar to untreated cells (note lines are parallel for polyglutamine and p38MAPK inhibition during this time interval). The later increase in inclusion formation when p38MAPK activity is inhibited is probably due to ROS generation via the aggregated protein. The computer model predicts that much higher levels of mRFP^u^ will be observed in PI treated cells than in untreated cells, as expected for a proteasome substrate (Figure 5C). Critically, inhibition of p38MAPK activity reduced the accumulation of mRFP^u^ in the computer model. Overall the simulations and experimental data are in good agreement, indicating that the model describes the important molecular relationships of the system as portrayed in Figure 4.

### Activation of p38MAPK contributes to ROS production but not direct proteasome inhibition

**Figure 6:**
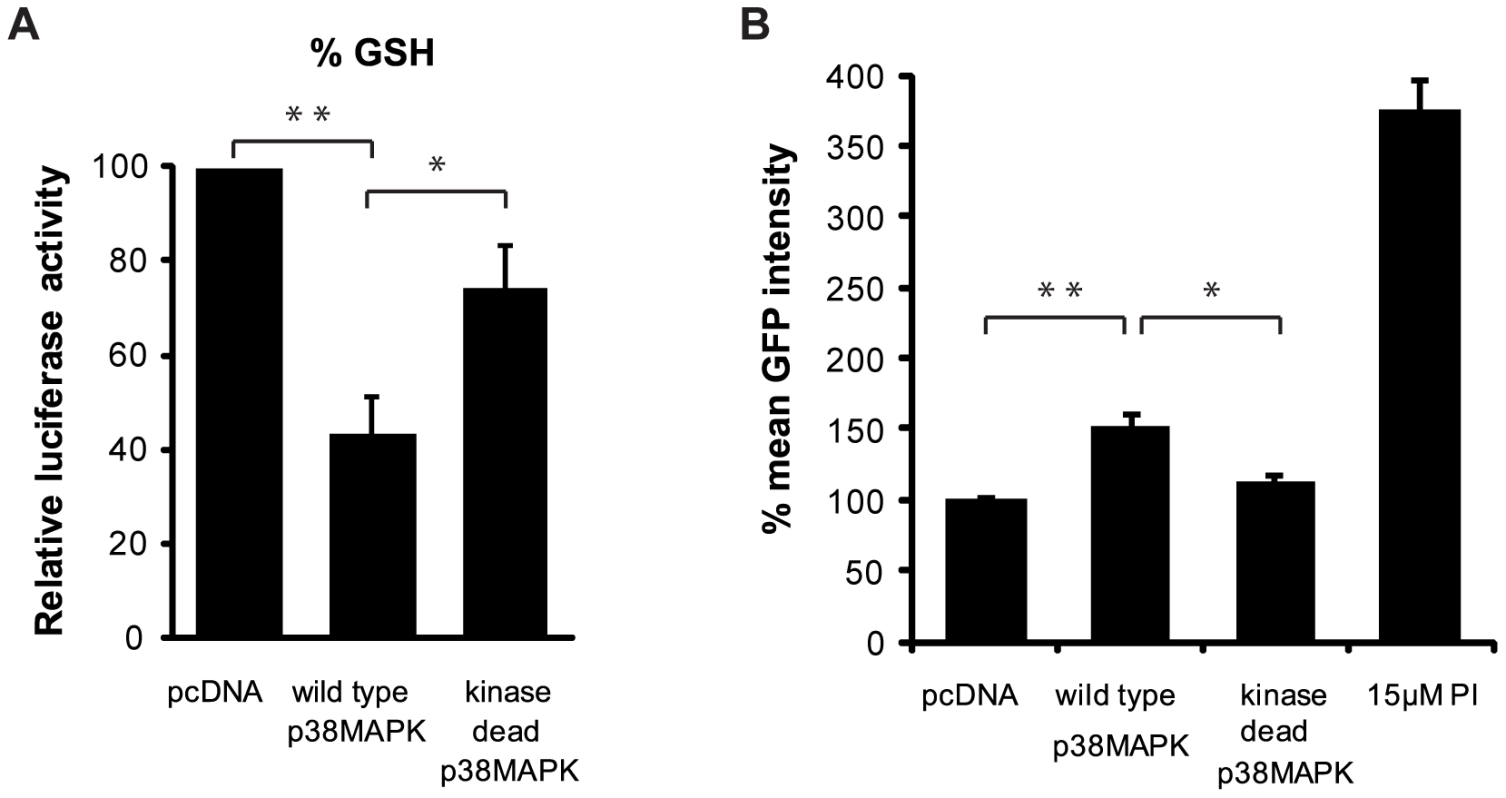
Effects of p38MAPK activity on GSH and GFP^u^ levels. A) Lysates from cells expressing either wild type p38MAPK, kinase dead p38MAPK, or pcDNA control plasmids were assayed for reduced GSH content 48 hours post transfection. Cells transfected with wild type p38MAPK had lower levels of reduced GSH when compared to the pcDNA empty vector control (**p<0.05) and kinase dead p38MAPK expressing cells (*p = 0.06). B) Activation of the p38MAPK pathway leads to proteasomal inhibition. Wild type p38MAPK or kinase dead p38MAPK were transfected into 3T3 cells stably expressing the proteasome reporter GFP^u^. Cells were collected at 48 hours post-transfection and GFP^u^ intensity was analyzed by flow cytometric analysis. Cells over-expressing wild type p38MAPK had significantly higher levels of GFP^u^ accumulation when compared to cells expressing kinase dead p38MAPK (*p<0.05) or pcDNA empty vector control (**p<0.01). PI treatment served as a positive control.

Based on our single-cell analysis data, we speculated that the activation of p38MAPK and UPS impairment were contributing to the production of reactive oxygen species (ROS) and that SKF86002 may be counteracting this cellular response. A genetic approach was adopted to test this hypothesis, utilizing transfection of cells with expression vectors encoding wild type or kinase dead versions of p38MAPK. Whereas pharmacological inhibition may affect multiple p38MAPK isoforms any modulation of cellular response observed with the genetic approach would be attributable to the alpha isoform of p38MAPK exclusively. We first confirmed that in our U87MG cell system the expression vectors were capable of modulating p38MAPK activity using phosphorylation of HSP27 as a proxy marker (Figure S1). Cells were then transfected with the p38MAPK expression vectors or with an empty vector control construct and lysates were assayed for reduced glutathione (GSH) content, a marker of oxidative status within cells. We found that overexpression of wild type p38MAPK resulted in a significant decrease in GSH levels whereas the GSH content in cells expressing kinase dead p38MAPK was less affected in comparison to controls (Figure 6A). To determine if overexpression of wild type p38MAPK resulted in dysfunction of the UPS, we transfected the p38MAPK expression constructs into a cell line stably expressing GFP^u^, a well characterized proteasome sensor [10]. For these experiments we made use of a stable NIH 3T3 cell line we had previously generated; the GFP^u^ reporter is expressed at a lower level in the stable line and does not accumulate as an artefact of transfection-mediated overexpression. By flow cytometric analysis, we found that cells overexpressing wild type p38MAPK exhibited the highest levels of GFP^u^ intensity whereas the levels of GFP^u^ in cells expressing the kinase dead p38MAPK or the empty vector were similar (Figure 6B). These data suggested that the activation of p38MAPK was negatively affecting UPS function. To test whether this inhibition of the proteasome was a direct affect of p38MAPK activation, we transfected cells with the same set of plasmids and assayed their proteasome activity using fluorogenic substrates. No significant differences were noted (Figure S1). Taken together, the data indicate that the expression of p38MAPK does affect the oxidative status of cells but does not directly inhibit the proteasome.

**Figure 7:**
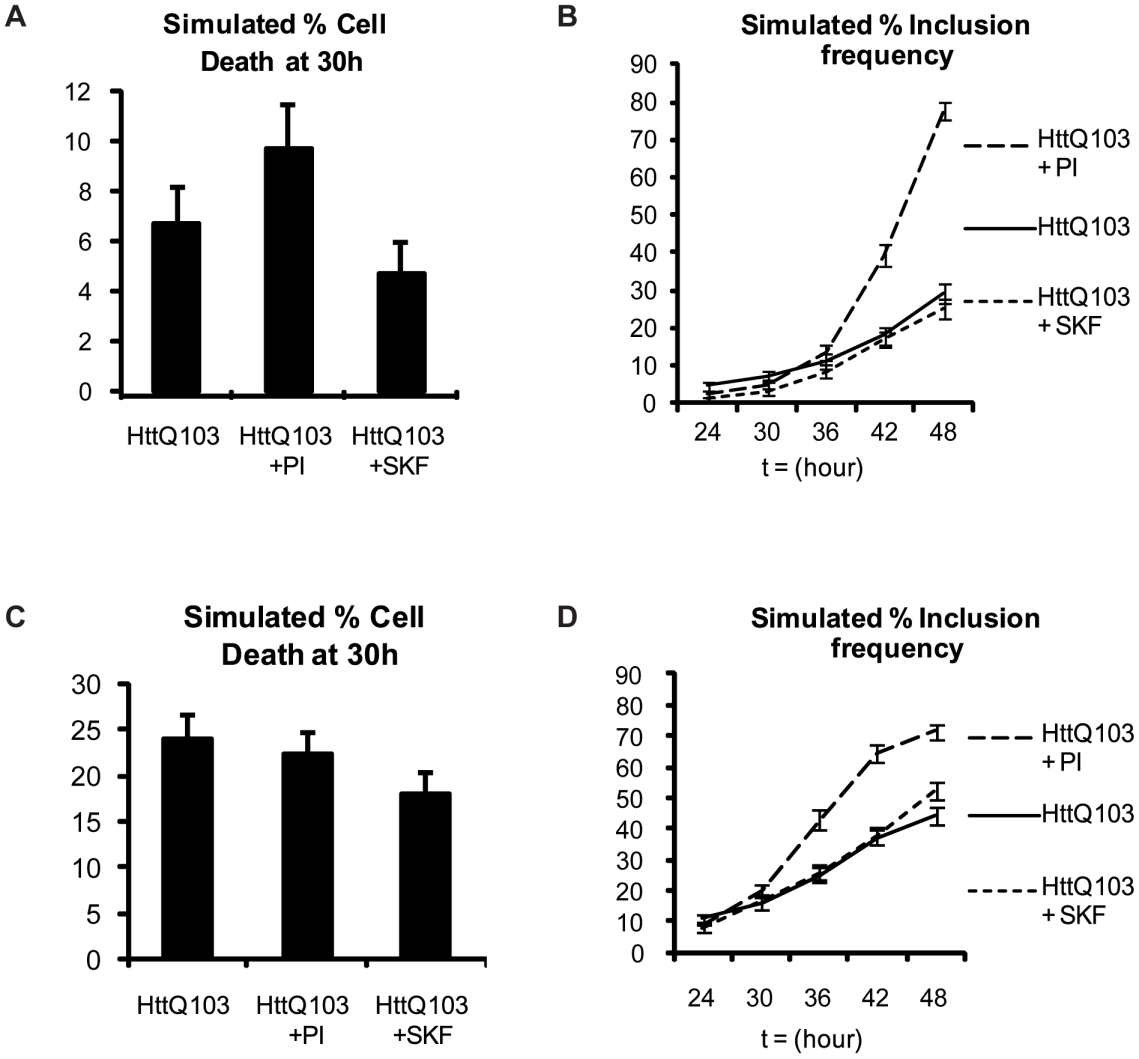
Simulated effects of p38MAPK activation without ROS generation. A) When the feedback loop is broken by eliminating ROS production the model predicts that cell death will be reduced under all conditions. B) Under ROS-free conditions the model predicts no significant difference in the numbers of inclusions at each time point between untreated polyglutamine-expressing cells or cells treated with a p38MAPK inhibitor. Similarly, no significant difference is predicted in the number of inclusions at early time points between untreated cells or cells treated with a proteasome inhibitor. At later time points (> = 42h) more inclusions are predicted for the PI treated cells due to the accumulation of misfolded protein which cannot be degraded. C) Model re-fitted to data of untreated poly-glutamine-expressing cells predicts more cell deaths but still no significant differences between treatments. D) Model re-fitted to data of untreated poly-glutamine-expressing cells shows no significant differences between untreated and treated cells at early time points. At 48h, the model predicts a small but significant increase in the number of inclusions for cells treated with p38MAPK inhibitor indicating that the model no longer fits the data. The error bars represent the standard error of the percentage given by 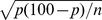 where n is the number of simulation runs (n = 300) and p is the percentage of cells.

Since aggregated protein leads to increased levels of ROS, inhibition of p38MAPK may simply delay cell death and thereby allow more aggregation to take place. To determine the predicted outcome should p38MAPK *not* be involved in generating more ROS we removed the reaction for ROS generation via p38MAPK from the model and repeated the computer simulations. Without p38MAPK-generated ROS less cell death was predicted under all conditions (Figure 7A). With the feedback loop broken in this way the model also predicted that there would be no significant difference in the numbers of inclusions at each time point between untreated cells and cells treated with a p38MAPK inhibitor (Figure 7B). We also refitted the model for HttQ103 without treatments and no feedback loop using the experimental data for cell death and inclusion formation (Figure 7C–D). Since the model predicted less cell deaths and lower levels of inclusions than the data, we increased the parameters for inclusion formation (*k_aggPolyQ_*) and cell death (*k_p38death_* and *k_PIdeath_*). We also increased the parameter for ROS generation via p38MAPK (*k_genROSp38_*) since this had the effect of increasing both the levels of inclusions and the number of cell deaths. We then ran the model with the treatment for proteasome inhibition and p38MAPK inhibition. The model was not able to reproduce the decline in cell death or the lower levels of inclusions when p38MAPK is inhibited (evident from the comparison of Figure 7C–D with Figure 5A–B). Therefore the model indicates that a feedback loop from p38MAPK to ROS is required to explain the experimental data.

## Discussion

**Figure 8:**
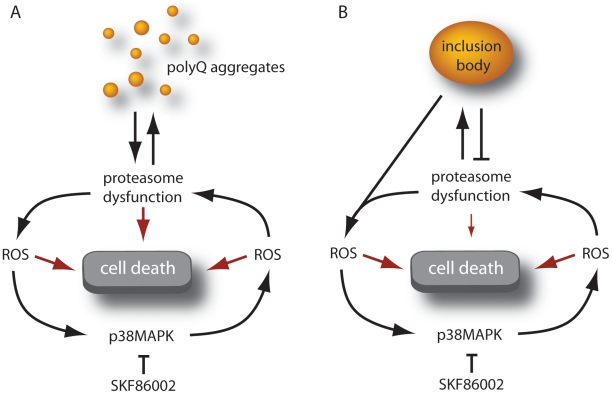
Proposed model of expanded-polyglutamine induced toxicity. A) Expression of expanded-polyglutamine proteins leads to proteasome inhibition, promoting the generation of ROS. This in turn leads to p38MAPK activation (which can be blocked by pretreatment of cells with SKF86002) and the further generation of ROS through a mechanism which is likely to involve mitochondria. An increased burden of oxidatively damaged proteins leads to further proteasome impairment, and yet more ROS generation. B) Proteasome impairment ultimately leads to inclusion formation, which temporarily reduces UPS dysfunction. IBs perpetuate ROS production through concentration of iron, ensuring continued activation of p38MAPK. Pharmocological inhibitors of p38MAPK such as SKF86002 can preclude the initiation of the vicious cycle or potentially interrupt it at a later stage.

Based on the experimental data and the computer simulations reported herein we propose a vicious cycle mechanism of polyglutamine-mediated cytotoxicity (Figure 8). In the proposed mechanism the initiating event is the inhibition of the proteasome by small aggregates of misfolded protein. It has been previously shown that proteasome inhibition leads to the generation of ROS (reviewed in [12]) and the activation of MKK3 and MKK6 kinases upstream of the stress kinase p38MAPK [13], [14]. Activation of p38MAPK by its upstream regulators may exacerbate the problem by promoting downstream ROS production (through a mechanism discussed below). By damaging other cellular proteins the reactive oxygen would provide an additional burden to the UPS, which is normally charged with the proteolytic degradation of damaged or abnormal proteins. Increasing proteasome inhibition would lead to further accumulation of misfolded proteins, ultimately coalescing into inclusion bodies. By reducing local concentrations of the small aggregates that are thought to be most inhibitory to the proteasome the IBs may temporarily alleviate proteasome inhibition, but by concentrating iron they may promote the further generation of ROS, ensuring yet more damage and the conditions that will sustain p38MAPK activation. Whether or not there is self-amplification of the vicious cycle as a consequence of increasing ROS production we postulate that it is sustained p38MAPK activity and proteasome inhibition that will ultimately lead to cell death.

The mathematical model was based on our previous model of the ubiquitin/proteasome system which we modified and extended to include turnover and aggregation of polyglutamine proteins. The model predictions were in close agreement with the experimental data indicating that the proposed network in Figure 4 captures the important components in the system. However, we found that there were some discrepancies between the model predictions and experimental data regarding cell death at early time-points under conditions of proteasome inhibition. This suggests that there is something missing from the model. Since proteasome inhibition affects all protein turnover, the missing part could be a pro-apoptotic protein. One such candidate is the transcription factor p53 which is normally rapidly turned over by the proteasome. A future extension of our current model to include a pro-apoptotic protein would be fairly straightforward as we have previously modelled the p53 system [15]. An advantage of modelling over laboratory experiments is that it is easy to manipulate the system on a computer, whereas the same experiments in the laboratory may be impossible or very costly to do. For example, although it would be difficult to prove experimentally that ROS generation by p38MAPK is required to explain the experimental data, the hypothesis could easily be tested in the mathematical model by simply removing the reaction of p38MAPK-dependent ROS generation and repeating the simulations. The model confirmed that p38MAPK is involved in generating more ROS since with the feedback loop broken the model output no longer fitted the experimental data.

We have previously shown that pharmacological blockade of p38MAPK can protect cells from polyglutamine-mediated cell death [9], and the current experimental data and mathematical modeling provide an explanation for the efficacy of this intervention: by breaking the vicious cycle the p38MAPK inhibitor precludes further damage and proteasome inhibition. The data we present herein support a central role for ROS in the proposed cycle of p38MAPK activation, proteasome inhibition, and protein aggregation that ultimately leads to cell death. The importance of ROS in protein misfolding disorders is not a new concept; indeed the cytotoxicity of elevated ROS generated by mutant huntingtin has been convincingly demonstrated by the laboratory of Rubinsztein [16]. Intriguingly, the proteasome is itself an important regulator of oxidative damage in neurons and proteasome inhibition is known to induce mitochondrial dysfunction and promote oxidative damage to DNA and protein (reviewed in [12]). Once some threshold of polyQ-mediated proteasome inhibition is reached it seems entirely plausible that a self-perpetuating loop of ROS generation and p38MAPK activation could lock the cell into a dysfunctional state. Indeed, a directly analogous positive feedback loop involving ROS and p38MAPK activation was recently proposed for the induction of senescence in mammalian cells. By combining bioinformatics, stochastic computer modeling, and direct experimental interventions Passos et al. have provided convincing evidence that sustained activation of p38MAPK is required for generation of mitochondrial ROS, which by generating DNA damage ensures the continued activation of p38MAPK in senescent cells [17]. The ROS generated as a consequence of proteasome inhibition also appears to be of mitochondrial origin [18], so the cascade of events in cells expressing misfolded protein may be very similar to that in cells in which the initiating event is exposure to ionizing radiation (as was the case in the Passos paper). If a similar loop were operating in cells expressing expanded polyglutamine proteins one might expect to find ROS-mediated DNA damage leading to activation of ATM and phosphorylation of histone H2AX, as has indeed been documented in cells from Huntington's disease and SCA-2 patients [19]. The activation of ATM and formation of H2AX repair foci appears to precede the formation of IB [20], but may be coincident with p38MAPK activation and the generation of ROS. If our model is correct pretreatment of cells with the p38MAPK inhibitor should reduce the number of H2AX foci in cells expressing HttQ103. We are currently testing this hypothesis.

Enhanced levels of autophagy may also contribute to the protective effects of the p38MAPK inhibitor we have observed. Although the effects may be dependent on cell type, there is evidence that the alpha isoform of p38MAPK inhibits autophagy [21] [note that this is the same isoform utilized in our genetic experiments, and may be the target of primary importance to all of the interventions described herein]. Low level inhibition of the proteasome is known to promote autophagy [22], but as proteasome inhibition increases the activation of p38MAPK may limit autophagic clearance of protein aggregates. Enhancement of autophagy has been proposed as a therapeutic strategy for the treatment of polyglutamine disorders such as Huntington's disease [23], and pharmacological blockade of p38MAPK may provide benefit by multiple mechanisms. It may break the vicious cycle of ROS generation while promoting autophagy-mediated clearance of aggregates in cells already compromised for proteasome function.

We have performed the current set of experiments in U87MG cells, a human glioblastoma cell line. These cells were chosen for their ease of handling, including their high transfection efficiency. Because the critical components of the proposed vicious cycle are universally present in mammalian cells (including p38MAPK, proteasomes, and ROS from mitochondria) it is likely that a self-perpetuating loop could be triggered by expanded polyglutamine proteins in any cell type, and we have previously demonstrated the protective effect of a p38MAPK inhibitor in normal and transformed cells of both human and mouse origin [9]. We nevertheless recognize that the kinetics of the events we have described may be markedly different for neurons *in situ*.

Although the short term benefit of IB formation (through improved proteasome function) has been demonstrated in cultured neurons [4], [5] the longer term implications of IB in neurodegenerative diseases must be considered. A transient alleviation by IB of the polyglutamine-mediated proteasome burden was recently demonstrated *in vivo* in an inducible model of Huntington's disease [24], but no long term impairment of proteasome function was observed in the brains of these mice. If the IB concentrates iron and promotes ROS generation through Fenton chemistry [8] the IB, once formed, might ensure the perpetuation of the vicious cycle proposed in Figure 3C. The failure to detect this effect in older mice expressing a polyQ protein may relate to the inability of the reporter system to detect less than a 40% decrease in proteasome inhibition [24] or may relate to differences between the mouse model and the human disease. In human polyglutamine disorders the disease may progress over years if not decades, and the later consequences of IB formation might therefore be more severe. Clinical evidence supports a deleterious role for IB in human disease. In polyglutamine repeat diseases such as HD, frontotemporal lobar degeneration, and RNA-mediated diseases such as myotonic dystrophy, inclusion-mediated titration of transcription factors (like CBP), tar DNA-binding protein 43 (TDP-43), and RNA splicing factors (for example muscleblind), respectively, may represent an important molecular mechanism of disease [6]. Added to these effects would be the deleterious effects we ascribe to the ROS-mediated vicious cycle.

## Materials and Methods

### Expression constructs

The pEGFP-N1 expression construct was purchased from Clontech (Palo Alto, California, USA). Htt-Q25 and Htt-Q103 expression constructs containing a synthetic insert encoding exon 1 or human Huntingtin containing a polyglutamine tract of either 25Q or 103Q fused to the yellow fluorescent reporter protein (YFP) were generous gifts from Dr. Ron Kopito (Stanford University). These are designated HttQ25YFP or HttQ103YFP. A red fluorescent proteasome reporter was generated by PCR-mediated transfer of the degron sequence from the GFP^U^ reporter (Bence et al.; the gift of Dr. Ron Kopito) to the C terminus of the monomeric red fluorescent protein (the gift of Dr. Robert Campbell, University of Alberta). Under normal conditions, mRFP^u^ is quickly degraded by the 26S proteasome, but during conditions of proteasomal impairment, turnover of mRFP^u^ is reduced, leading to an accumulation of mRFP^u^ that is visible by fluorescent microscopy. To simultaneously express the expanded YFP-tagged polyglutamine proteins and the red fluorescent proteasome reporter the former was inserted into NheI site upstream of the internal ribosome entry site (IRES) in the vector pIRES (Clontech, Palo Alto, California, USA) and the latter was inserted between the Xba I and Sal I sites downstream of the IRES element. The wild type and kinase dead p38MAPK variants were generous gifts from Dr. J. Han (The Scripps Research Institute, La Jolla, CA). The hyper-active p38MAPK construct was a gift from Dr. Oded Livnah (The Hebrew University of Jerusalem).

### Cell culture and transfections

The human U87MG glioblastoma cells (a gift from Dr. I. Lorimer at the Ottawa Hospital Research Institute) were maintained in Dulbecco's modified Eagle's medium (DMEM) and supplemented with 10% FBS and maintained in a 37°C incubator with 5% CO_2_. For transient transfections, cells were plated in either 96- or 6 well dishes 24hours prior to transfections. Subsequently, they were transfected using GeneJuice Transfection Reagent (Novagen, Madison, WI, USA) as per the supplier's protocol. 0.5µg or 3.0µg of plasmid DNA was used in each well of a 96 or 6 well dish. For p38MAPK inhibition experiments, cells were pre-treated for 2h with 20µM SKF86002 (Calbiochem) prior to transfection with various expression constructs.

### Western blot analysis

U87MG cells were harvested in protein lysis buffer consisting of 100mM Tris pH 6.8, 20mM DTT, 4% SDS, 5% glycerol. Protein concentrations were determined using the Bradford assay reagents (Bio-Rad, Hercules, CA, USA). Reduced proteins were resolved on a 10% SDS-polyacrylamide gel and electro-blotted onto a Hybond C nitrocellulose membrane (Amersham Bioscience Corp, Baie d'Urfé, QC). The membranes were stained with Ponceau S prior to immunoblotting with phospho-HSP-27 (polyclonal rabbit), total HSP-27 (rabbit polyclonal) (Cell Signaling, Danvers, MA), or Actin (Sigma-Aldrich). Proteins were detected using the HRP method and SuperSignal West Pico Chemiluminescent Substrate reagents (Pierce, Rockford, IL, USA). Proteins were visualized using the GeneGnome (Syngene, Frederick, MD, USA).

### Survival assays

Cell viability was assessed by flow cytometry using propidium iodide exclusion. Adherent and non-adherent cells were transfected with various constructs for 30 hours, harvested and stained with Propidium Iodide. For p38MAPK inhibition experiments, cells were pre-treated for 2h with 20µM SKF86002 (Calbiochem) prior to transfection with various expression constructs. For proteasome inhibition experiments, cells were treated with Proteasome Inhibitor I (Calbiochem) 24h post-transfection at a final concentration of 25µM. Fluorescent detection was analyzed by flow cytometry using a Beckman Coulter Quanta SC MPL. Data and analysis were done using Quanta Analysis software (Beckman Coulter, Inc., Brea, CA, USA).

### Live-cell imaging

75 000 U87MG glioblastoma cells were seeded onto a Delta T4 culture dish system (Bioptechs, Butler, PA) and maintained in a 37°C incubator with 5% CO_2_ for 24h hours. Cells were transfected with 2ug of plasmid DNA encoding HttQ103YFP-pIRES-mRFP^u^ for 24 hours before being transferred onto a heated stage maintained at 37°C and at 5% CO_2_ using a Delta T4 culture dish temperature controller and cell perfusion system (Bioptechs, Butler, PA). For p38MAPK inhibition experiments, cells were pre-treated for 2h with SKF86002 for a final concentration of 20µM to preclude kinase activation upon transfection. For proteasome inhibition experiments, cells were treated with Proteasome Inhibitor I (Calbiochem) 24h post-transfection at a final concentration of 25µM (treatment with PI prior to transfection is not possible due to its immediate toxicity). For buthionine sulphoximide (BSO)-induced depletion of glutathione experiments, cells were treated 24h post-transfection with BSO (Sigma) 24h post-transfection at a final concentration of 5mM. Microscopy was performed 24 hours post-transfection on a Zeiss Axiovert 200M inverted fluorescent microscope for a total of 24 hours. Fully automated multidimensional acquisition was controlled using Axiovision 4.8 software. Images were acquired using a 10× objective (EC Plan-Neofluar) with a side-mounted AxiocamHRm camera. Yellow fluorescent protein or red fluorescent protein was excited using the Zeiss Colibri LED illumination system (LEDmodule 505nm or LED module 590nm) and detected using the appropriate filters (46HEYFP or 61HEGFP/HcRED, respectively). Fixed exposure times were as follows: Brightfield phase contrast 1ms; YFP 100ms; RFP 188ms. Images were taken at 10 minute intervals for 48 hours and compiled into video files using Axiovision 4.8 software (Carl Zeiss, Thornwood, NY).

### Glutathione assay

Cell lysates from U87MG cells over-expressing wild type p38MAPK or kinase dead p38MAPK were analyzed for reduced GSH content using a luciferase kit (GSH-Glo) from Promega (Madison, WI). 10,000 cells were seeded in 96-well plates and transfected with 0.5µg of DNA for 48 hours. Cells were collected and analyzed for GSH following the manufacture's protocol.

### Proteasome inhibition analysis

Mouse NIH 3T3 cells were co-transfected with GFP^u^ and a PGK-driven puromycin resistance gene (gift of Dr. M. McBurney, Ottawa Hospital Research Institute). Cells stably expressing the proteasome reporter GFP^u^ were selected over 2 weeks in a final concentration of 2.0µg/ml. For proteasome assays, wild type p38MAPK, kinase dead p38MAPK, or pcDNA were transfected into 3T3-GFP^u^ cells for 48 hours. Cells were collected and analyzed for GFP^u^ expression by flow cytometry (Beckman Coulter Quanta SC MPL). Mean GFP intensity was analyzed using the Quanta Analysis software and subsequently graphed using Excel (Microsoft).

### Statistical analysis

Statistical significance was determined by a two tailed Student's t-test. Unless otherwise indicated, values were considered significant when p<0.05.

### Mathematical model

The model was developed to mimic the experimental system so that simulations could be performed to see which parameters affected the different cellular outcomes. The model was initially fitted to experimental data where HttQ103 had been added to cells but without any inhibitors. The model was then used to mimic the experimental treatments and the model predictions were compared to the experimental results. If there were discrepancies between the model predictions and experimental results, the model was modified (either by changing parameter values or by the addition of further reactions). The model was then re-run for the experiment without any treatments to check that it still fitted the experimental data. If the model did not fit, further adjustments were made and the procedure repeated. We originally started with a model that did not contain a feedback loop from p38MAPK to ROS but found that it was necessary to include this loop in order to get the model to fit both the data for the treatment with p38MAPK inhibitor and the data without the treatment. Further details are given in Text S1.

The model was encoded in the Systems Biology Markup Language (SBML) as this standard allows models to be easily shared, modified and extended [25]. SBML is a way of representing a network of interactions so that it can be simulated on a computer and the evolution of the system over time can be followed. SBML shorthand was used to create the SBML code which was then converted into full SBML [26]. The network diagram is given in Figure 4, and Tables S1 and S2 in Text S1 give details of the species and reactions respectively. Text S1 also contains more detail of events and parameter values (Tables S3 and S4 in Text S1). Since there is large variability in cellular outcomes in terms of both inclusion formation and cell death, we used stochastic simulation. This was based on the Gillespie algorithm [27] which assumes that collisions of molecules occur within a reaction vessel and that at most only two molecules can collide. We chose this method of simulation as there are low copy numbers of many of the species and random effects play a major role in this model, as can be seen by the cell to cell variability of the model output. It should be noted that we have a few reactions which have more than two molecules in the list of reactants. These are the reactions for the aggregation of polyQ where we assume that ROS affects the reaction kinetics although ROS itself is not consumed by the reaction (and so also appears in the list of products). We use a function of ROS in the kinetic law so that we have a pseudo second order reaction rather than a third order reaction. Although this may seem to be a violation of the assumptions of the Gillespie algorithm, this provides a simple way to allow for the effects of ROS on the aggregation process rather than adding many more reactions and parameters. The model is available from the Biology of Ageing e-Science Simulation and Integration (BASIS) system ([28], [29]) and the Biomodels database (ID:MODEL1002250000) [30].

### Model Assumptions

We assume that the addition of the polyQ gene to the cell resulted in continuous synthesis of the polyQ protein. It is also degraded by the proteasome so that total levels remain fairly constant with a half-life of about 20 hours [31]. We set the levels of polyQ sythesis and degradation so that the half-life would be 20 hours if the proteasome did not become inhibited by aggregates. Two molecules of polyQ interact to form a small aggregate (AggPolyQ1). We assume that ROS affects the aggregation kinetics if it rises above basal levels. The aggregate can grow in size by the addition of further polyQ proteins in a reversible manner. However, when the aggregate reaches a certain threshold size, we assume that disaggregation can no longer take place and that instead an inclusion forms (SeqAggP). This threshold represents the seed and is assumed to be of size six based on data for amyloid fibril polymerization [32]. Since mutant huntingtin forms amyloid-like filaments, it is reasonable to assume that it has similar aggregation kinetics [33], [34]. A very recent study shows that mutant huntingtin forms three major pools: monomers, oligomers and inclusion bodies [35]. Interestingly, the study showed that the pool of oligomers as a proportion of total huntingtin did not change over a time period of 3 days despite continued conversion of monomer to inclusion bodies. We also compared the levels of oligomers (represented by the species AggPolyQ[i], where i = 1–5) in our simulation output in cells which formed inclusion bodies (Figure S2) and discuss the results in the Text S1 section. It has been shown that small aggregates bind to proteasomes and inhibit proteasomal function [36]. Therefore, we assume that AggPolyQ can bind to the proteasome and so reduce the pool of available proteasomes. However we assume that inclusions do not interfere with the degradation machinery. We also include a species to represent mRFPu and assume that this is turned over with a half-life of about 30 minutes [36]. We assume that ROS is continuously generated and removed with a half-life of 1 hour [37] but that basal levels are low (about 10 molecules). We assume that small aggregates may generate ROS, so that the level of ROS is dependent on the amount of small aggregates (either bound to the proteasome or free pools). We also assume that the presence of inclusions will increase levels of ROS but with a much smaller effect than small aggregates. We represent p38MAPK in two forms: unphosphorylated (p38) and phosphorylated (p38-P) with p38-P being the active state. We assume that high levels of ROS activate p38MAPK and that high levels of p38-P initiate a signalling cascade that results in cell death. We set the rate of this reaction so that it is unlikely to occur when p38-P levels are low and the probability of the reaction occurring increases with increasing levels of p38-P. However, since the model is stochastic, it is possible that even low levels of p38-P will occasionally signal for cell death. We also assume that if the level of proteasomes bound by aggregates increases above a threshold of about 50%, then another signalling pathway leads to cell death due to the accumulation of the pro-apoptotic protein p53. As in the p38 death pathway, cell death due to aggregates inhibiting the proteasome may occur even when levels of AggP Proteasome are fairly low. The reactions for the cell death pathways are shown in Table S2 in Text S1. After cell death occurs, a dummy parameter *k_alive_* is set to zero to prevent further reactions occurring, and a dummy species to record the cause of cell death is set to 1. This makes it possible to plot the time of cell death, the cause of death and to count the number of cell deaths of each type in multiple simulations.

We also assume that proteasomes bound by AggP, polyQ or mRFPu may be sequestered into inclusions if degradation does not take place. If AggP is sequestered into inclusions, then this will help alleviate the increase in ROS due to protein aggregation, since we assume that small aggregates lead to greater ROS generation than inclusions.

We also include a generic pool of protein (NatP) which can misfold to become (MisP). We assume that misfolded protein can be either refolded, ubiquitinated and degraded or at high concentrations it may start to aggregate. Once an inclusion forms, misfolded protein may be sequestered into the inclusion body, including MisP bound to proteasomes.

## Supporting Information

Figure S1 A)Western blot analysis with the phospho-HSP-27 antibody of cell extracts from U87MG cells co-transfected with pcDNA empty vector, wild type p38 MAPK, kinase dead p38MAPK, or hyper-active p38 MAPK expression constructs in cells expressing HttQ25 or HttQ103. The analysis revealed that phospho-Hsp27 levels were reduced in extracts from cells co-transfected with kinase dead p38MAPK and showed a complete abrogation of HSP-27 phosphorylation in cells treated with SKF86002. The antibody raised against total HSP-27 was used to detect total HSP-27 levels and actin served as a loading control. B) Expression of p38 MAPK does not directly inhibit the proteasome. Cells were transfected with either wild type p38 MAPK, kinase dead p38MAPK, or pcDNA control plasmids and assayed for their ability to process a peptidylglutamyl- or chymotrypsin-specific fluorogenic substrate. Cells were lysed 48 hours post-transfection and assayed in triplicates. The relative activity of the proteasome was measured 12 hours after the addition of the substrates. PI added to lysates was used as a control to demonstrate the specificity of the PI inhibitor for the chymotrypsin-like activity of the proteasome. Data was normalized to the proteasome activity in lysates from cells transfected with a pcDNA control vector. Experiments were performed in triplicate. Error bars represent standard deviation of the mean.
(0.28 MB TIF)

Figure S2 Distribution of polyQ monomers, oligomers and inclusion bodies. Simulation output from 3 runs of the model showing that the size of the oligomeric pool remains constant even when inclusions form.
(1.26 MB TIF)

Text S1 Model details.
(0.14 MB DOC)

Video S1 Time-lapse imaging of IB formation and UPS dysfunction in U87MG cells transfected with HttQ103YFP-pIRES-mRFP^u^. Live cell imaging was initiated at 24 hours post-transfection and images were acquired every 10 minutes. Cells were visualized under white light, and filters that detect YFP or RFP. The brightfield and fluorescence emanating from the YFP and RFP channels were merged to create a movie file. Images were acquired using a 10× objective for a total of 24 hours. At the beginning of the movie HttQ103 is expressed throughout the cell. At 36 hours (the half way point) many cells have formed an inclusion body and have notable accumulation of the red reporter protein (indicative of proteasome inhibition). Note that based on their morphology many cells with IB appear to be viable at the end of the movie.
(5.79 MB MOV)

Video S2 Time-lapse imaging of IB formation and UPS dysfunction in U87MG cells transfected with HttQ103YFP-pIRES-mRFP^u^. Live cell imaging was initiated at 24 hours post-transfection and images acquired every 10 minutes. Cells were visualized using filters that detect YFP or RFP. Fluorescent images were merged to create a movie file. Images were acquired using a 10× objective for a total of 24 hours. At the beginning of the movie HttQ103 is expressed throughout the cell. At 36 hours (the half way point) many cells have formed an inclusion body and have notable accumulation of the red reporter protein (indicative of proteasome inhibition). Note that based on their morphology many cells with IB appear to be viable at the end of the movie.
(5.63 MB MOV)

Video S3 Time-lapse imaging of U87MG cells transfected with HttQ103YFP-pIRES-mRFP^u^ treated with PI 24 hours post-transfection showing an increase of IB formation and UPS dysfunction. Cells were visualized under white light, and filters that detect YFP or RFP. The brightfield and fluorescence emanating from the YFP and RFP channels were merged to create a movie file. Images were acquired every 10 minutes using a 10× objective for a total of 24 hours. The movie shows rapid and progressive accumulation of the mRFP^u^ reporter protein (red colour) after the addition of PI. This coincides with the formation of IB at an earlier time point (30 hours). At the conclusion of the movie there are considerably more IB and cell death events as compared to untreated cells (videos S1 and S2).
(6.83 MB MOV)

Video S4 Time-lapse imaging of U87MG cells expressing HttQ103YFP-pIRES-mRFP^u^ treated with PI 24 hours post-transfection. Cells were visualized using filters that detect YFP or RFP. Fluorescence emanating from the YFP and RFP channels was merged to create a movie which shows an increase of IB formation and UPS dysfunction. Images acquired every 10 minutes using a 10× objective for a total of 24 hours. The movie shows rapid and progressive accumulation of the mRFP^u^ reporter protein (red colour) after the addition of PI. This coincides with the formation of IB at an earlier time point (30 hours). At the conclusion of the movie there are considerably more IB and cell death events as compared to untreated cells (videos S1 and S2).
(4.26 MB MOV)

Video S5 Time-lapse imaging of U87MG cells transfected with HttQ103YFP-pIRES-mRFP^u^ treated with BSO 24 hours post-transfection. Treated cells displayed an increase in UPS dysfunction without IB formation. Cells were visualized under white light and filters that detect YFP or RFP. The brightfield and fluorescence emanating from the YFP and RFP channels was merged to create a movie file. Images were acquired every 10 minutes using a 10× objective for a total of 24 hours. Cells treated with BSO display a rapid and progressive accumulation of the mRFP^u^ reporter protein (red colour), but the frequency of IB formation is not notably different than in untreated cells (videos S1 and S2).
(4.76 MB MOV)

Video S6 Time-lapse imaging of U87MG cells transfected with HttQ103YFP-pIRES-mRFP^u^ treated with BSO 24 hours post-transfection showing an increase in UPS dysfunction without IB formation. Cells were visualized using filters that detect YFP or RFP. Fluorescence emanating from the YFP and RFP channels was merged to create a movie file. Images were acquired every 10 minutes using a 10× objective for a total of 24 hours. Cells treated with BSO display a rapid and progressive accumulation of the mRFPu reporter protein (red colour), but the frequency of IB formation is not notably different than in untreated cells (videos S1 and S2).
(1.85 MB MOV)

Video S7 Time-lapse imaging of U87MG cells transfected with HttQ103YFP-pIRES-mRFP^u^ pre-treated with SKF86002 two hours prior to transfection. The movie shows a decrease in IB formation and UPS dysfunction. Live cell imaging was initiated at 24 hours post-transfection, with images acquired every 10 minutes. Cells were visualized under white light, and filters that detect YFP or RFP. The brightfield and fluorescence emanating from the YFP and RFP channels were merged to create a movie file. Images were acquired using a 10× objective for a total of 24 hours. Throughout the movie there are fewer cells with IBs (relative to untreated cells in movies S1 and S2). The levels of the mRFPu reporter protein (red colour) remain lower than in untreated cells and do not increase until late in the movie.
(5.95 MB MOV)

Video S8 Time-lapse imaging of U87MG cells transfected with HttQ103YFP-pIRES-mRFP^u^ pre-treated with SKF86002 two hours prior to transfection. The movie shows a decrease in IB formation and UPS dysfunction. Live cell imaging was initiated at 24 hours post-transfection and images were acquired every 10 minutes. Cells were visualized using filters that detect YFP or RFP. Fluorescence emanating from the YFP and RFP channels was merged to create a movie file. Images were acquired using a 10× objective for a total of 24 hours. Throughout the movie there are fewer cells with IBs (relative to untreated cells in videos S1 and S2). The levels of the mRFPu reporter protein (red colour) remain lower than in untreated cells and do not increase until late in the movie.
(1.17 MB MOV)
