# Supplementary figures and images for: Experimental and Computational Analysis of Polyglutamine-Mediated Cytotoxicity
Source: PLoS Comput Biol. 2010 Sep 23;6(9):e1000944. doi: 10.1371/journal.pcbi.1000944 (PMC2944785; doi:10.1371/journal.pcbi.1000944)

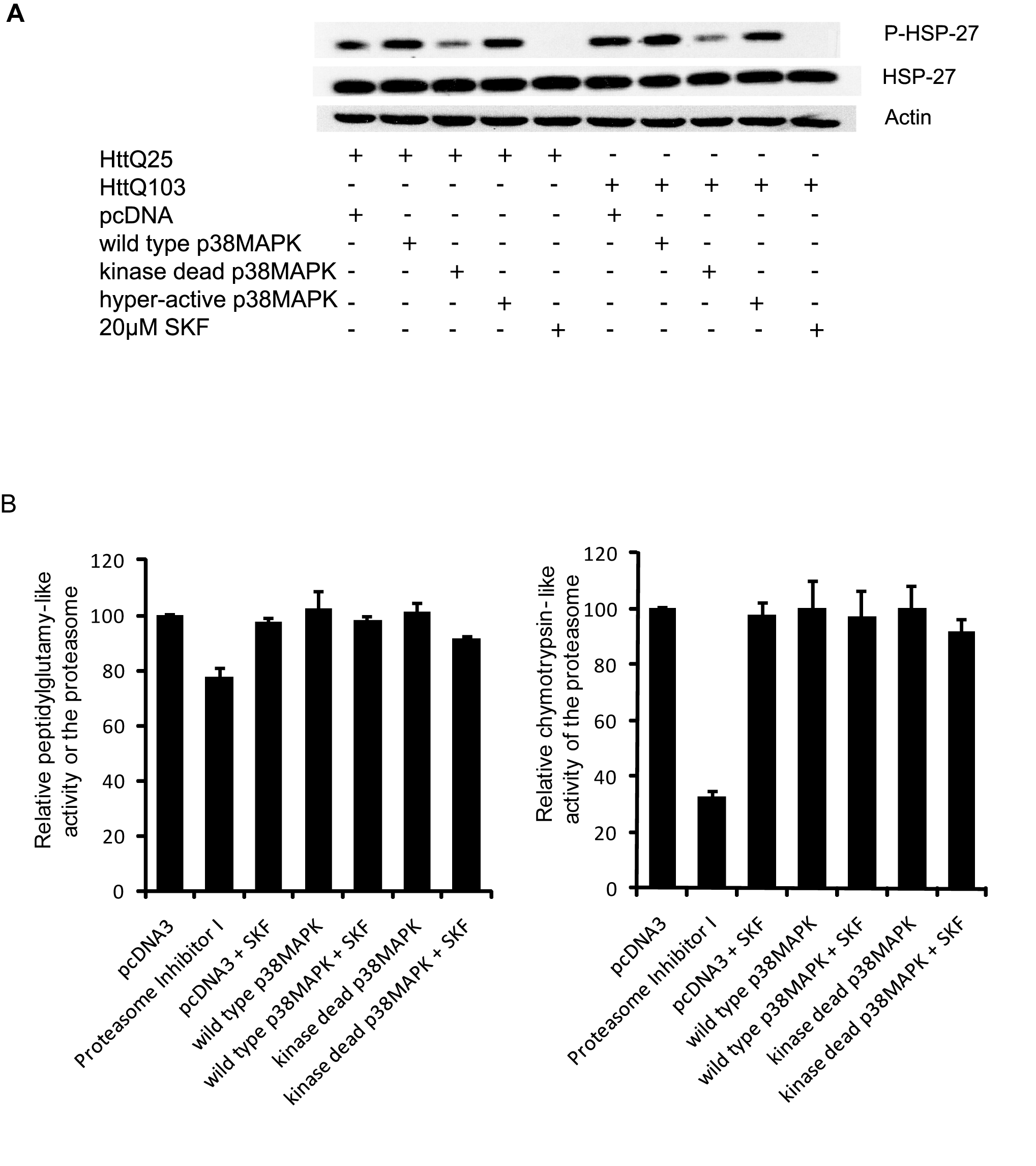

Supplement: Figure S1 — A)Western blot analysis with the phospho-HSP-27 antibody of cell extracts from U87MG cells co-transfected with pcDNA empty vector, wild type p38 MAPK, kinase dead p38MAPK, or hyper-active p38 MAPK expression constructs in cells expressing HttQ25 or HttQ103. The analysis revealed that phospho-Hsp27 levels were reduced in extracts from cells co-transfected with kinase dead p38MAPK and showed a complete abrogation of HSP-27 phosphorylation in cells treated with SKF86002. The antibody raised against total HSP-27 was used to detect total HSP-27 levels and actin served as a loading control. B) Expression of p38 MAPK does not directly inhibit the proteasome. Cells were transfected with either wild type p38 MAPK, kinase dead p38MAPK, or pcDNA control plasmids and assayed for their ability to process a peptidylglutamyl- or chymotrypsin-specific fluorogenic substrate. Cells were lysed 48 hours post-transfection and assayed in triplicates. The relative activity of the proteasome was measured 12 hours after the addition of the substrates. PI added to lysates was used as a control to demonstrate the specificity of the PI inhibitor for the chymotrypsin-like activity of the proteasome. Data was normalized to the proteasome activity in lysates from cells transfected with a pcDNA control vector. Experiments were performed in triplicate. Error bars represent standard deviation of the mean. (0.28 MB TIF) [file pcbi.1000944.s001.tif]

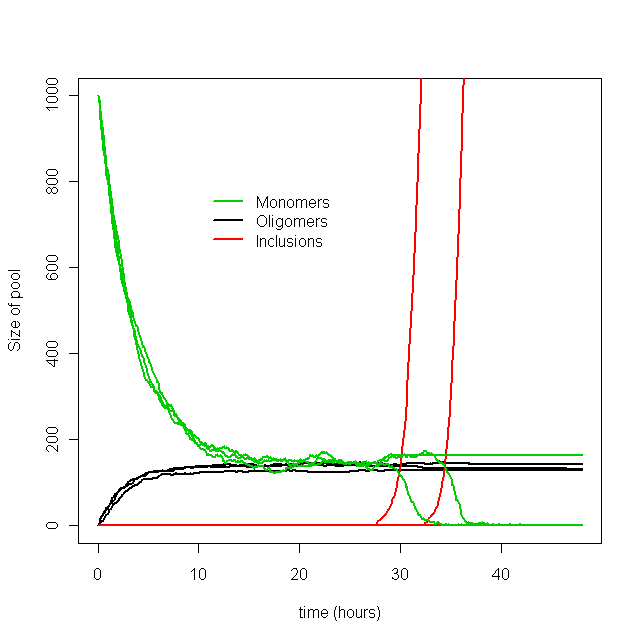

Supplement: Figure S2 — Distribution of polyQ monomers, oligomers and inclusion bodies. Simulation output from 3 runs of the model showing that the size of the oligomeric pool remains constant even when inclusions form. (1.26 MB TIF) [file pcbi.1000944.s002.tif]
