# Supplementary material for: Experimental and Computational Analysis of Polyglutamine-Mediated Cytotoxicity
Source: PLoS Comput Biol. 2010 Sep 23;6(9):e1000944. doi: 10.1371/journal.pcbi.1000944 (PMC2944785; doi:10.1371/journal.pcbi.1000944)
Supplement: Text S1 — Model details. (0.14 MB DOC) [file pcbi.1000944.s003.doc]

**Supplementary Material: Details of Stochastic Computer Model**

**Fitting the model to experimental data.**

The model has 47 parameters but only a small set of these were used to fit the model to experimental data. These parameters are listed in Table 4. The table also indicates which parameters effect aggregation kinetics, cell death or mRFPu levels. We began by fitting the model under normal conditions (addition of PolyQ without any other treatements) to the time course data for inclusion formation. Since the data corresponds to proportions of many cells, we ran the model 100 times initially and calculated the proportion of “simulated cells” with inclusions at the same time points. This was repeated many times until the best set of parameters was found. The parameters for cell death were adjusted to fit the experimental data in a similar way to the aggregation kinetic parameters. Finally we used the kinetic data for the levels of mRFPu. In order to establish the level of mRFPu detection, we ran the model without PolyQ as under these conditions mRFPu is undetectable. The mean level of mRFPu is 300 but due to random fluctuations it can vary between 200 and 400. So we set a value of 400 as the threshold for detection.

After finding the best fit of the model to the data of untreated cells we mimicked the experiments of inhibiting the proteasome at 24h by adding an event to the model (see Table 3). We then compared the model outcome with the experimental data. Originally the model did not closely match this data and so we adjusted parameters that affected inclusion formation and cell death, concentrating on those that were particularly affected by the proteasome e.g. rate of proteasome binding by aggregates. When we obtained a reasonable match we then redid the simulations with simulated untreated cells to check. Finally we validated the model with data for p38MAPK inhibition. In the initial stages of model building we did not include a feedback loop from p38MAPK to ROS i.e. the reaction for ROS generation by p38MAPK was not included. However, we found that we could not initially fit the model to the data with p38MAPK. A recent paper showed that a feedback between p38MAPK and ROS is necessary for cell senescence [1] and so we decided to try and include this reaction in our current model. This enabled us to fit the data for all experimental conditions. In order to verify that this additional reaction is necessary, we also fitted the model without the loop to the experimental data for the addition of HttQ103 without any treatments and then ran the model for the inhibition experiments (Figure 7).

The model fitting was quite time-consuming to do but we plan to use a more automated approach in future using Bayesian approaches with the Calibayes system which has been developed at Newcastle University [2]. Currently the system handles fitting model parameters to data for single cell time course experiments but it will be possible to adapt the software to handle population kinetic data.

**Parameter values not used for model fitting**.

The majority of parameter values were obtained from the literature or based on previous models and as they were not directly involved in aggregation or cell death pathways we did not use these to fit the model. We will now give a brief description of these parameters.

The parameters for the turnover of PolyQ and mRFPu were set according to their half-lives which we assumed to be 20 hours and 30 minutes respectively when the proteasome is not inhibited. We assume that PolyQ is more difficult for the proteasome to degrade than mRFPu so that *k_degPolyQ_* < *k_degmRFPu_*. The parameter which has most affect on the half-life is the rate of binding of the protein to the proteasome (*k_binPolyQProt_* and *k_binmRFPuProt_*). We set the synthesis rate of each protein to zero and adjusted the parameters for proteasome binding to achieve the desired half-life. Then we set the parameters for protein synthesis so that protein levels remain constant over time (there will be variability in the levels due to stochastic simulation but the mean level is constant). We also include a pool of generic protein which may misfold and either be refolded or degraded. We assumed that half-life of this protein pool was about 10 hours, that refolding and degradation occurred at about the same order of magnitude with slightly more refolding taking place. With these assumptions we were able to set the parameters for misfolding, refolding, binding to the proteasome and degradation. We also assumed that misfolded protein could aggregate but only if levels of misfolded protein are high, so that the rate of aggregation is set very low (three orders of magnitude lower than PolyQ). We assume that there is basal turnover of ROS and set the parameters for generation and removal of ROS so that the half-life is about 1 hour and the mean level of basal ROS (under conditions of no aggregation) is 10.

There are three dummy parameters in the model. First we include a parameter called *k_alive_* in every reaction so that when cell death occurs the value of this parameter is changed from 1 to zero. This prevents any further reactions occurring after a cell death. The parameter *k_proteff_*  is present in all the protein degradation reactions and is initially set to 1. The value of this parameter is reduced by a SBML event (see Table S3) to mimic a reduction in proteasome efficiency when proteasome inhibitor is added. Finally the parameter *k_p38ac_*_t_ allows p38MAPK activity to be reduced when p38 inhibitor is added. In the computer experiments for p38MAPK inhibition we set *k_p38ac_*_t_=0.05 at the beginning of the simulation.

**Comparison of model output to data by Olshina et al. [3]**

A study has recently been published in which the authors tracked mutant huntingtin aggregation in cells using sedimentation velocity analysis. They found that mutant huntingtin formed three major pools: monomers, oligomers and inclusion bodies. Interestingly, they found that the oligomer pool in proportion of total huntingtin did not change over 3 days although monomers continued to be converted to inclusion bodies. In our model, monomers of huntingtin are represented by the species PolyQ, oligomers by AggPolyQ[i] (i=1-5) and inclusion bodies by SeqAggP. Therefore we selected cells from our simulations and tracked the levels of each pool over time and used the sum of the AggPolyQ[i]s to represent the pool of oligomers. As in the experimental data, we also found that after the initial increase in levels of AggPolyQ (since it was initially set to zero), levels remained fairly constant over time and were similar even when the pool of monomers was depleted and inclusion bodies formed (Figure S1). The reason for the invariant size of the oligomeric pool in the model is that we assumed that once inclusion bodies have formed, monomers could be directly sequestered into these, whereas the oligomers were unable to be sequestered in this way. We based this assumption on a number of aggregation studies and kinetic models which suggest that inclusion bodies grow in size by the addition of monomers although other mechanisms have been proposed [4]. Therefore, oligomers persist whether or not inclusion bodies are formed and these are the most toxic species since they interfere with the cellular machinery. Olshina et al [3] found that the chaperone Hsc70 decreases oligomeric pools by increasing the flux of monomers to inclusion bodies. We could extend our current model to include the role of chaperones to examine this further. Since we have already built an SBML model of the chaperone system [5], this would be fairly straightforward to do.

Table S1 List of Species

| Species description | Name | Database term | Initial amount |
| --- | --- | --- | --- |
| Polyglutamine-containing protein | PolyQ | P42858 | 1000 |
| red fluorescent protein | mRFPu | - | 300 |
| 26S Proteasome | Proteasome | GO:0000502 | 1000 |
| PolyQ bound to proteasome | PolyQ_Proteasome | - | 0 |
| mRFPu bound to proteasome | mRFPu_Proteasome | - | 0 |
| PolyQ aggregate of size i (i= 1-5) | AggPolyQi | - | 0 |
| Inclusion | SeqAggP | - | 0 |
| Aggregated protein bound to proteasome | AggP_Proteasome | - | 0 |
| Reactive oxygen species | ROS | CHEBI:26523 | 10 |
| Active p38MAPK | p38_P | Q16539 | 0 |
| Inactive p38MAPK | p38 | Q16539 | 100 |
| Generic pool of native protein | NatP | - | 19,500 |
| Misfolded protein | MisP | - | 0 |
| Misfolded protein bound to proteasome | MisP_Proteasome | - | 0 |
| Small aggregate of size i (i= 1-5) | AggPi | - | 0 |
| Dummy species to record cell death due to proteasome inhibition | PIdeath | - | 0 |
| Dummy species to record cell death due to p38MAPK activation | p38death | - | 0 |

Table S2 List of reactions

| No.^a^ | Reaction name | Reaction | Rate law^b^ | Parameter value |
| --- | --- | --- | --- | --- |
| 1 | PolyQ synthesis | Source *→* PolyQ | *k_synPolyQ_* | 7.0 E-3 molecule s^-1^ |
| 2 | PolyQ/proteasome binding | PolyQ + Proteasome *→*  PolyQ_Proteasome | *k_binPolyQ_*[#PolyQ][#Proteasome] | 5.0 E-8 molecule^-1^ s^-1^ |
| 3 | PolyQ/proteasome release | PolyQ_Proteasome *→* PolyQ + Proteasome | *k_relPolyQ_*[#PolyQ_Proteasome] | 1.0 E-9 s^-1^ |
| 4 | PolyQ degradation | PolyQ_Proteasome *→*  Proteasome | *k_degPolyQ_*k_proteff_* [#PolyQ_Proteasome] | 2.5 E-3 s^-1^ |
| 5 | mRFPu synthesis | Source *→* mRFPu | *k_synmRFPu_* | 1.38 E-1 molecule s^-1^ |
| 6 | mRFPu / proteasome binding | mRFPU + Proteasome *→* mRFPu_Proteasome | *k_binmRFPu_*[#mRFPu][#Proteasome] | 5.0 E-7 molecule^-1^ s^-1^ |
| 7 | mRFPu / proteasome release | mRFPu_Proteasome *→* mRFPU + Proteasome | *k_relmRFPu_*[#mRFPu_Proteasome] | 1.0 E-8 s^-1^ |
| 8 | mRFPu degradation | mRFPu_Proteasome *→* Proteasome | *k_degmRFPu_*k_proteff_* [#mRFPu_Proteasome] | 5.0 E-3 s^-1^ |
| 9 | PolyQ aggregation1 | 2PolyQ+ROS *→* AggPolyQ1+ROS | *k_aggPolyQ_*[#PolyQ][#PolyQ-1]*  ([#ROS^2^]/(10^2^+[#ROS^2^])) | 5.0 E-8 molecule^-1^ s^-1^ |
| 10 | PolyQ aggregation[i+1] (i=1-4) | AggPolyQi+PolyQ+ROS *→* AggPolyQ(i+1)+ROS | *k_aggPolyQ_*[#AggPolyQi][#PolyQ]*  ([#ROS^2^]/(10^2^+[#ROS^2^])) | 5.0E-8 molecule^-1^ s^-1^ |
| 11 | PolyQDisaggregation1 | AggPolyQ1*→*2AggPolyQ | *k_disaggPolyQ1_*[#AggPolyQ1] | 5.0E-7 s^-1^ |
| 12 | PolyQDisaggregation2 | AggPolyQ2*→*AggPolyQ1  +PolyQ | *k_disaggPolyQ2_*[#AggPolyQ2] | 4.0E-7 s^-1^ |
|  | PolyQDisaggregation3 | AggPolyQ3*→*AggPolyQ3  +PolyQ | *k_disaggPolyQ3_*[#AggPolyQ3] | 3.0E-7 s^-1^ |
|  | PolyQDisaggregation4 | AggPolyQ4*→*AggPolyQ3  +PolyQ | *k_disaggPolyQ4_*[#AggPolyQ4] | 2.0E-7 s^-1^ |
|  | PolyQDisaggregation5 | AggPolyQ5*→*AggPolyQ4  +PolyQ | *k_disaggPolyQ5_*[#AggPolyQ5] | 1.0E-7 s^-1^ |
| 13 | Inclusion formation | AggPolyQ5+PolyQ *→ 7*SeqAggP | *k_aggPolyQ_*[#AggPolyQ5][#PolyQ] | 5.0 E-8 molecule^-1^ s^-1^ |
|  | Inclusion growth | SeqAggP + PolyQ *→* 2SeqAggP | *k_seqPolyQ_*[#SeqAggP][#PolyQ] | 8.0 E-7 molecule^-1^ s^-1^ |
| 14 | Proteasome inhibition  by aggregates[i] (i=1-5) | AggPolyQi + Proteasome *→* AggP_Proteasome | *k_inhprot_*[#AggPolyQ[i]] [#Proteasome] | 5.0 E-9 molecule^-1^ s^-1^ |
| 15 | Basal ROS production | Source *→* ROS | *k_genros_* | 1.7 E-3 molecule s^-1^ |
| 16 | ROS removal | ROS *→* Sink | *k_remros_*[#ROS] | 2.0 E-4 s^-1^ |
| 17 | ROS generation AggPolyQ[i] (i=1-5) | AggPolyQ[i]*→*AggPolyQ[i]+ ROS | *k_genrosAggP_* [#AggPolyQ[i]] | 5.0 E-6 s^-1^ |
| 18 | ROS generation AggP_Proteasome | AggP_Proteasome *→* AggP_Proteasome + ROS | *k_genrosAggP_*[#AggP_Proteasome] | 5.0 E-6 s^-1^ |
| 19 | p38MAPK activation | ROS + p38 *→* ROS + p38_P | *k_actp38_*[#ROS][#p38] | 5.0 E-6 molecule^-1^ s^-1^ |
| 20 | p38MAPK inactivation | p38_P *→* p38 | *k_inactp38_*[#p38_P] | 2.0 E-3 s^-1^ |
|  | AggP_Proteasome sequestering | AggP_Proteasome+SeqAggP*→*2SeqAggP | *k_seqAggPProt_*[#AggP_Proteasome]  [#SeqAggP] | 5.0E-7 molecule^-1^ s^-1^ |
|  | PolyQ_Proteasome sequestering | PolyQ_Proteasome+SeqAggP*→*2SeqAggP | *k_seqPolyQProt_*[#PolyQ_Proteasome]  [#SeqAggP] | 5.0e-7  molecule^-1^ s^-1^ |
|  | Protein synthesis | Source *→* NatP | *k_synNatP_* | 2.4 molecule s^-1^ |
|  | Protein misfolding | NatP+ROS*→*MisP+ROS | *k_misfold_*[#NatP][#ROS] | 2.0E-6 molecule^-1^ s^-1^ |
|  | Protein refolding | MisP*→*NatP | *k_refold_*[#MisP] | 8.0E-5 s^-1^ |
|  | MisP/Proteasome binding | MisP+Proteasome*→* MisP_Proteasome | *k_binMisPProt_*[#MisP][#Proteasome] | 5.0E-8 molecule^-1^ s^-1^ |
|  | MisP_Proteasome release | MisP_Proteasome*→*  MisP+Proteasome | *k_relMisPProt_*[#MisP_Proteasome] | 1.0E-8 s^-1^ |
|  | Degradation of misfolded protein | MisP_Proteasome*→* Proteasome | *k_degMisP_*k_proteff_* [#MisP_Proteasome] | 1.0E-2 s^-1^ |
|  | MisP aggregation | 2MisP *→* AggP1 | *k_aggMisP_*[#MisP][#MisP-1]/2.0 | 1.0E-11 molecule^-1^ s^-1^ |
|  | MisP aggregation [i] (i=1-4) | AggP[i] + MisP *→* AggP[i+1] | *k_agg2MisP_*[#MisP][#AggP[i]] | 1.0E-10 molecule^-1^ s^-1^ |
|  | MisP disaggregation1 | AggP1*→*2MisP | *k_disaggMisP1_*[#AggP1] | 5.0E-7 s^-1^ |
|  | MisP disaggregation2 | AggP2*→*AggP1+MisP | *k_disaggMisP2_*[#AggP2] | 4.0E-7 s^-1^ |
|  | MisP disaggregation3 | AggP3*→*AggP2+MisP | *k_disaggMisP3_*[#AggP3] | 3.0E-7 s^-1^ |
|  | MisP disaggregation4 | AggP4*→*AggP3+MisP | *k_disaggMisP4_*[#AggP4] | 2.0E-7 s^-1^ |
|  | MisP idsaggregation5 | AggP5*→*AggP4+MisP | *k_disaggMisP5_*[#AggP5] | 1.0E-7 s^-1^ |
|  | MisP Inclusion formation | AggP5 + MisP *→* 7SeqAggP | *k_agg2MisP_*[#AggP5][#MisP] | 1.0E-10 molecule^-1^ s^-1^ |
|  | MisP Inclusion growth | SeqAggP+ MisP *→* 2SeqAggP | *k_seqmisp_*[#SeqAggP][#MisP] | 1.0E-9 molecule^-1^ s^-1^ |
|  | MisP_Proteasome sequestering | MisP_Proteasome+SeqAggP*→*2SeqAggP | *k_seqMisPProt_*[#MisP_Proteasome]  [#SeqAggP] | 5.0e-7  molecule^-1^ s^-1^ |
|  | mRFPu_Proteasome sequestering | mRFPu_Proteasome+SeqAggP*→*2SeqAggP | *k_seqmRFPuProt_*[#mRFPu_Proteasome]  [#SeqAggP] | 5.0e-7  molecule^-1^ s^-1^ |
|  | mRFPu sequestering | mRFPu+SeqAggP*→*2SeqAggP | *k_seqPolyQProt_*[#mRFPu][#SeqAggP] | 1.0e-10  molecule^-1^ s^-1^ |
|  | Proteasome inhibition AggP[i] (i=1-5) | AggP[i]+Proteasome*→* AggP_Proteasome | *k_inhprot_*[#AggP[i]][#Proteasome] | 5.0E-9 molecule^-1^ s^-1^ |
|  | ROS generation AggP[i] (i=1-5) | AggP[i] *→* AggP[i] + ROS | *k_genROSAggP_*[#AggP[i]] | 5.0E-6 s^-1^ |
| 21 | p38 ROS generation | p38_P*→*p38_P+ROS | *k_genROSp38_*k_p38act_*[#p38_P] | 7.0E-4 s^-1^ |
|  | SeqAggP ROS generation | SeqAggP*→*SeqAggP+ROS | *k_genROSSeqAggP_*[#SeqAggP] | 1.0E-7 s^-1^ |
| 22 | P38 cell death | p38_P*→*p38_P+p38death | *k_p38death_*k_p38act_* [#p38_P] | 9.0E-8 s^-1^ |
| 23 | PI cell death | AggP_Proteasome*→* AggP_Proteasome+PIdeath | *k_PIdeath_*[#AggP_Proteasome] | 2.5E-8 s^-1^ |

^a^Numbers refer to reactions shown in Figure S1. ^b^All rate laws contain a dummy parameter *k_alive_*=1. ^c^*k_proteff_*=1 is a dummy parameter to allow proteasome efficiency to be reduced when proteasome inhibitor is added. . ^d^*k_p38ac_*_t_=1 is a dummy parameter to allow p38 activity to be reduced when p38 inhibitor is added.

Table S3 List of events

| Event name | Trigger | Assignments |
| --- | --- | --- |
| PI Cell Death | PIdeath>0 | *k_alive_* = 0 |
| p38 Cell Death | p38death>0 | *k_alive_* = 0 |
| Proteasome Inhibition | t>86400.0 | *k_proteff_*=0.05 |

Table S4 List of parameters used for fitting the model

| Parameter name | Experimental data used in model fitting | | |
| --- | --- | --- | --- |
|  | % Cell Death | % Inclusion bodies | mRFPu levels |
| *k_aggPolyQ_* |  | x |  |
| *k_seqPolyQ_* |  | x |  |
| *k_inhprot_* | x |  | x |
| *k_genrosAggP_* | x | x |  |
| *k_actp38_* | x |  |  |
| *k_seqAggPProt_* |  | x |  |
| *k_seqPolyQProt_* |  | x |  |
| *k_genROSp38_* | x | x |  |
| *k_p38death_* | x |  |  |
| *k_PIdeath_* | x |  |  |

**References**

1. Passos JF, Nelson G, Wang C, Richter T, Simillion C, et al. (2010) Feedback between p21 and reactive oxygen production is necessary for cell senescence. Mol Syst Biol 6: 347.

2. Chen Y, Lawless C, Gillespie CS, Wu J, Boys RJ, et al. (2010) CaliBayes and BASIS: integrated tools for the calibration, simulation and storage ofbiological simulation models. Briefings in Bioinformatics in press.

3. Olshina MA, Angley LM, Ramdzan YM, Tang J, Bailey MF, et al. (2010) Tracking mutant huntingtin aggregation kinetics in cells reveals three major populations including an invariant oligomer pool. Journal of Biological Chemistry 285: 21807-21816.

4. Morris AM, Watzky MA, Finke RG (2009) Protein aggregation kinetics, mechanism, and curve-fitting: A review of the literature. Biochimica et Biophysica Acta (BBA) - Proteins & Proteomics 1794: 375-397.

5. Proctor CJ, Soti C, Boys RJ, Gillespie CS, Shanley DP, Wilkinson DJ,et al. (2005) Modelling the actions of chaperones and their role in ageing. Mech Ageing Dev 126:119-131.
